# A biocatalytic peptidobiosensing molecular bridge for detecting osteosarcoma marker protein

**DOI:** 10.3389/fchem.2022.1112111

**Published:** 2023-01-12

**Authors:** Pengwei Jing, Ying Wang, Weixue Sun, Guishi Li, Zuofu Zhang, Qiang Xu, Hao Li

**Affiliations:** ^1^ Articulation Surgery and Sport Medicine Ward, Yantai Yuhuangding Hospital, Yantai, China; ^2^ Department of Otolaryngology Head and Neck Surgery, Yantai Yuhuangding Hospital, Yantai, China; ^3^ School of Biological Science and Technology, University of Jinan, Jinan, China

**Keywords:** peptidosensor, osteosarcoma maker protein, tyrosine cross-linking, three in one probe, enzyme-free detection

## Abstract

A biosensing scheme requiring only one-step sample incubation before signal collection, and using a compact “three-in-one” probe of target-binding, signal conversion, and amplification, may greatly simplify the design of biosensors. Therefore, sparing the multi-step addition of enzymes, protein, and nanomaterial, as well as the associated complexity and non-specific interactions. In this work, a peptide probe aimed at such compact features has been designed, based on protein-triggered, conformation-driven, and Cu (II) facilitated side-chain di-tyrosine cyclization. This design can use target-probe recognition to induce discriminated cross-linking and self-cleavage of the probe, resulting in retention or dissociation of a signal amplification motif from the search and consequently quantitative detection performance. The method has also been tested preliminarily in fractioned osteosarcoma clinical samples, showing an acceptable coherence between signal readout and clinical diagnosis. On the basis of these early findings, it is reasonable to assume that the proposed probe will be beneficial for the next development of tumor screening and prognosis sensors.

## Introduction

Proteins are among the important targets of therapeutic, biomedical, and bioanalytical researches (Borrebaeck, 2017; Otto and Sicinski, 2017; Schneider et al., 2017). Conventionally, proteins are targeted using antibodies, and protein-antibody interactions are converted to signal readout by enzymes. Recent developments have refined this method and added nanomaterial to improve the efficiency of signal enhancement. However, antibodies and enzymes, as well as other biomacromolecules, often present on their surface at various sites of different chemo-physical feature, liable to interfering interactions, also adding uncertainty to the interactions with nanomaterial of sizeable surface-to-volume ratio. Adding to this complexity is the fact that many biomacromolecules are employed as reagents, added step-by-step after the initial protein-probe interaction. Therefore a series of reactions of different spatiotemporal features takes place in the biosensing system. These characteristics tend to make the biosensing interactions as complicated as that in actual cellular life, where a large number of proteins are engaging in dynamic interplays. This complexity of the living system of protein interactions may explain that it is not unusual for biomedical research to arrive at contradictory and confusing conclusion on newly discovered interactions. But it is these interactions that compose the molecular mechanism of pathological processes, so the elucidation of these protein interactions is essential to future therapeutic and biomedical efforts. It becomes clear that the bioanalytical tools for detecting proteins need to be simpler, introducing the most minor complexity to the already complicated target protein interactions.

On how to simplify the biosensing system, inspiration could come from the living system of protein interactions where the non-covalent interplays between proteins are transient. If similar non-covalent molecular recognition of proteins can be designed to induce covalent cross-coupling, then protein interactions can become affixed and yield a more definite analytical readout (Koniev and Wagner, 2015; Sheng et al., 2015). Moreover, employed as biosensing elements, biomacromolecules that present a mosaic of different chemical sites naturally tend to invite the interference of weak but numerous non-specific interactions. If artificial targeting agents such as aptamers and peptides are utilized (Song et al., 2008; Zhang et al., 2017), their definite and straightforward chemical structure may reduce, to a great extent, such interference and complexity. Besides, simple artificial targeting ligands may also enable the design of a “compact probe” in which target-binding, signal conversion, and amplification can all be packed together, sparing the multi-step addition of reagents that accounts for the second source of complexity and interference. Such a covalent and reagent-less protein assay may prove more feasible and robust for future in-field applications.

**SCHEME 1:**
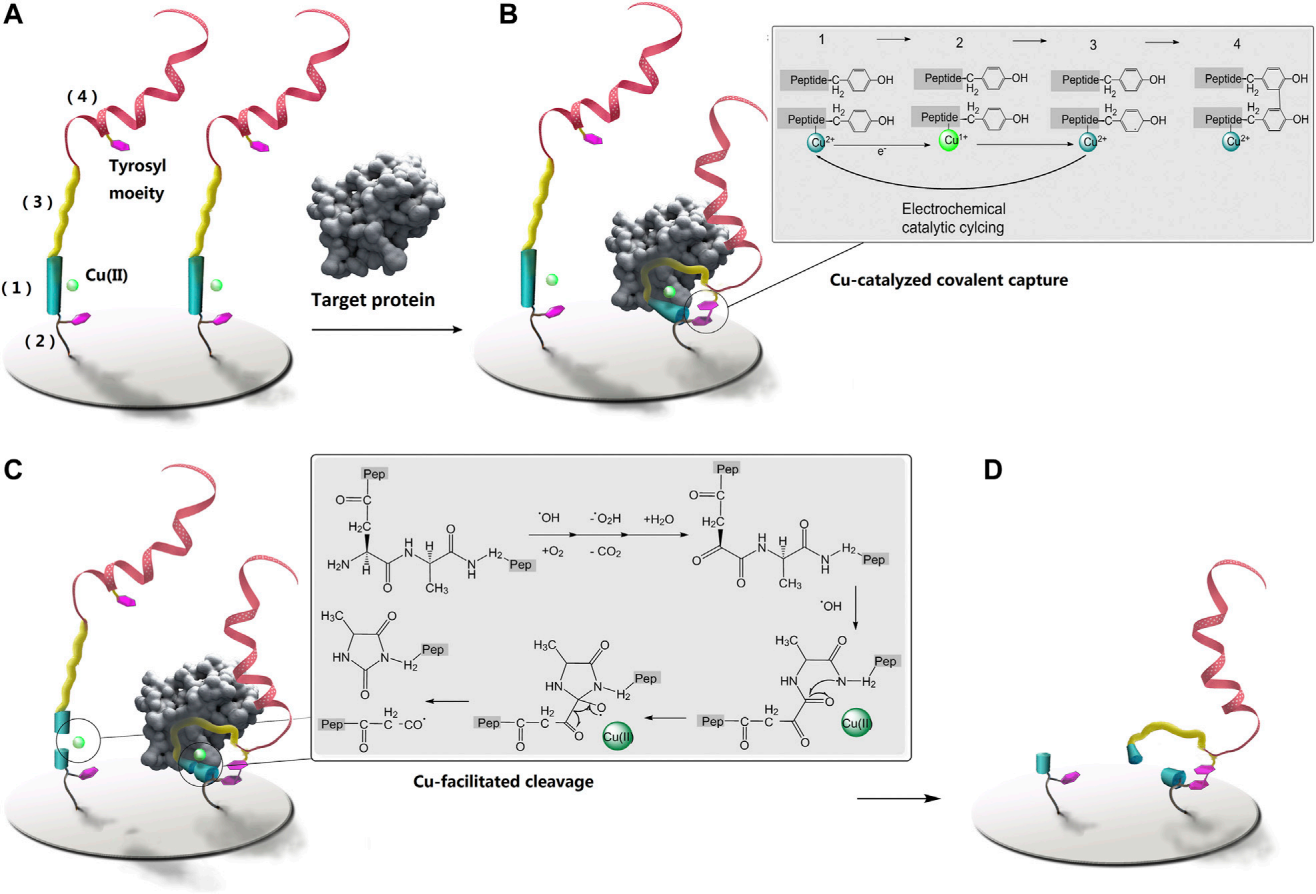
The proposed method for protein detection using protein-triggered, conformation-driven, Cu (II) facilitated side-chain di-tyrosine cyclization of the peptide probe. They were not drawn to scale. The two inset schemes display the coupling and cleavage processes. **(A)** is the sensing surface before sensing, **(B)** is covalent capturing of the target protein, **(C)** is covalent breaking of protein-free probe, and is the sensing surface after rinsing.

Based on these considerations we have designed a peptide-based covalent and reagent-less protein biosensing probe with enzyme-free signal enhancement. The reactivity of tyrosine (Wong and Wessel, 2008) and Cu ion (Skounas et al., 2010) under an electrochemically controlled oxidizing and reducing microenvironment has been enlisted to enable this new design. A linear peptide probe is designed to be of four motifs (Scheme 1A): (1) an N-terminal DAHK motif complexed with Cu ion (Hureau et al., 2011), (2) a tyrosine-containing and flexible poly-G linker, derived at the side chain of D in the DAHK the motif, immobilizing the probe onto the biosensing surface, (3) a protein targeting sequence to the C-terminal of DAHK the motif, and (4) the C-terminal poly tyrosine (40–200 moieties) as signal amplifier using electrochemical oxidation of the significant numbers of tyrosyl residuals. Upon electrochemical activation, DAHK-Cu can catalyze oxidative cross-linking of tyrosine moieties (Li et al., 2016) (framed sub-scheme on the right of Scheme 1b). The tyrosine moieties in the motif (2) and (4) are separated by the targeting sequence, a long sequence, so their spontaneous cross-linking can be prevented by the large entropy obstacle of the whole strand folding back onto itself (Scheme 1B, left). Protein binding may confine the protein targeting motif (3) in a more stable and curved-back conformation, facilitating the cross-linking (Scheme 1B, right), or the di-tyrosine cyclization reaction between tyrosine in poly tyrosine motif (4) and that in motif (2). While catalyzing the cross-linking, the N-terminal DAHK motif is also destroyed by Cu-catalyzed oxidative cleavage (Lee and Oe, 2016; Li et al., 2017) (Scheme 1C, detailed reaction process as shown in the framed sub-scheme to the right). Therefore all protein-free probes will lose their poly-tyrosine motif (4) (Scheme 1C, left), leaving only the motif (4) of protein-bound probes cross-linked with the stocks of the probe (Scheme 1C, right for signal amplification (Scheme 1D). This method has been applied in the quantification of several non-homologous proteins, as well as in clinical samples of hepatocellular carcinoma, with satisfactory analytical performance for different targets and in clinical settings. These results may promise the future application of the proposed methods for low-cost and robust protein detection in clinical practice. The proposed method generates fluorophore directly, rather than using quenching mechanisms, promising greatly lowered background and high sensitivity. This design has been demonstrated using a marker protein and therapeutic target of osteosarcoma, (Shi et al., 2019), integrin, which has been involved in various pathological signaling during the progress towards osteosarcoma (Vihinen et al., 1996; Guo et al., 2014; Liu et al., 2016). As integrin signaling often involves G protein (Wang et al., 2019), this protein has also been included as a relevant target, while avidin is selected as a reference target to demonstrate the versatility of the proposed method.

## Materials and methods

### Chemical and biological reagents

Synthetic peptide probes (theirs sequences was shown as Supplementary Figure S1), and a control synthetic peptide with the protein targeting sequence swapped as TSFAEYWNLLSP, were tailor-made as freeze-dried powder, with higher than 95% nominal purity, by Conngbibio Co., Ltd. Poly-tyrosine was purchased from Sigma-Aldrich. Recombinant G-protein subunit alpha and avidin were from Abcam, while recombinant integrin was from the R&D system. Recombinant human murine double minute 2 (hMDM2) (>98%) was purchased from ProSpec-Tany TechnoGene Ltd. The other compounds were all of an analytical caliber. The peptide probe solutions were made by combining 20 μM of the powder with 10 mM of phosphate buffer saline (PBS) (pH 7.4), and the Cu ion complexed peptide solutions were prepared by mixing the above stock solution at a 1:1 ratio with cupric chloride and then dialyzed against a blank PBS overnight. Stock solutions of the target proteins were prepared by dissolving the lyophilized powder of avidin and integrin. All of the solutions were created using double-distilled water and then purified using a Milli-Q purification system to a specific resistance of 18 M cm. After receiving approval from the local ethical committee, resected samples from osteosarcoma patients at Shandong University’s Qilu Hospital were obtained. The tissue samples were fractionated using an AbNova nucleus extraction kit in accordance with the manufacturer’s instructions.

### Electrode treatment

Transparent Au slides were trimmed to meet the cuvette size required for fluorescence measurement (about 100 Au layer condensed on ITO slides using vacuum evaporation method). These slides were washed with a gentle stream of high purity nitrogen before being submerged in the assembly solution (5 M probe and 5 mM Tris(2-carboxyethyl)phosphine hydrochloride (TCEP) in 10 mM PBS, pH 7.4) at 4°C for 16 h. The slides were then placed in a 9-mercaptoethanol (MN) solution (1 mM MN in 10 mM PBS, pH 7.4) at room temperature for 3 h.

### Detection

Protein samples were incubated with the slides for about 1 h at 37°C, followed by cyclic voltammetric scanning (beyond this range, large over-potential might result in apparent amino acid modification and protein denaturation). The slides were then thoroughly SDS rinsed.

### Measurements

A MicroCal ITC200 System was used to make measurements using isothermal titration calorimetry (ITC) (GE healthcare life sciences). At 25°C, the titration was carried out. A minimum 120 s gap separated each of the 38 1 L infusions that made up the titration protocol. Each set of data was adjusted for heats of dilution, which were calculated by titrating past saturation. Prior to titration, all solutions underwent degassing. Using software called Origin 7.0, the data were examined. A QM-4/2005 fluorescence spectrometer (Photon Technology International, Inc., Birmingham, NJ) fitted with a xenon lamp was used to measure the fluorescence emission spectra of surfaces. The detector and this light source were perpendicular to one another in the same plane. For fluorescence measurements, the slides were stored in a water-filled cuvette at a 60-degree angle from the cuvette’s base. The gold-coated surface was kept facing the detector rather than the light source. Tyrosine has an activating peak wavelength of 325 nm, whereas 3,4-Dihydroxyphenylalanine (DOPA) has a peak wavelength of about 360 nm. An Autolab ESPRIT system (Echo Chemie B.V., Netherlands) outfitted with a 670 nm monochromatic p-polarized light resource was used to conduct the SPR measurements. On a CHI660D Potentiostat (CH Instruments), electrochemical measurements were made using a traditional three-electrode setup: The working electrode is the modified electrode, the reference electrode is a saturated calomel electrode (SCE), and the counter electrode is platinum wire. In 10 mM PBS, pH 7.4, deoxygenated by purging with nitrogen gas, and maintained in this inert atmosphere during the electrochemical studies, square wave voltammograms (SUVs) were recorded. The experimental EIS settings were as follows: frequency range, .1 Hz–10 kHz; bias potential, .224 V vs. SCE; amplitude, 5 mV. 5 mM Fe(CN) 63/4 electrolyte solution with 1 M KCl. Error bars are displayed in the figures. The results were gathered from an independent experiment that was repeated at least three times. Using a JASCO J-750 circular dichroism spectrometer, circular dichroism (CD) spectra were acquired at wavelengths and scan rates between 190, 260, and 500 nm/min, respectively.

## Results

The proposed method is as illustrated in Scheme 1; its design has been demonstrated using three types of non-homogeneous proteins: integrin (α1β1), G protein subunit α1 (Gα1), and avidin, the targeting peptides (Meyer et al., 2006; Millward et al., 2007; Mas-Moruno et al., 2010) (their sequences as in Supplementary Figure S1) towards these three model proteins all tend to adopt a curved conformation when binding, favoring the proposed mechanism to work. The binding of these protein-targeting sequences in the designed probe has been first validated using isothermal titration calorimetry (ITC) to record the kinetics of peptide-protein binding. (Supplementary Figure S2), and relatively strong 1:1 binding can be observed for the tested probes.

**FIGURE 1:**
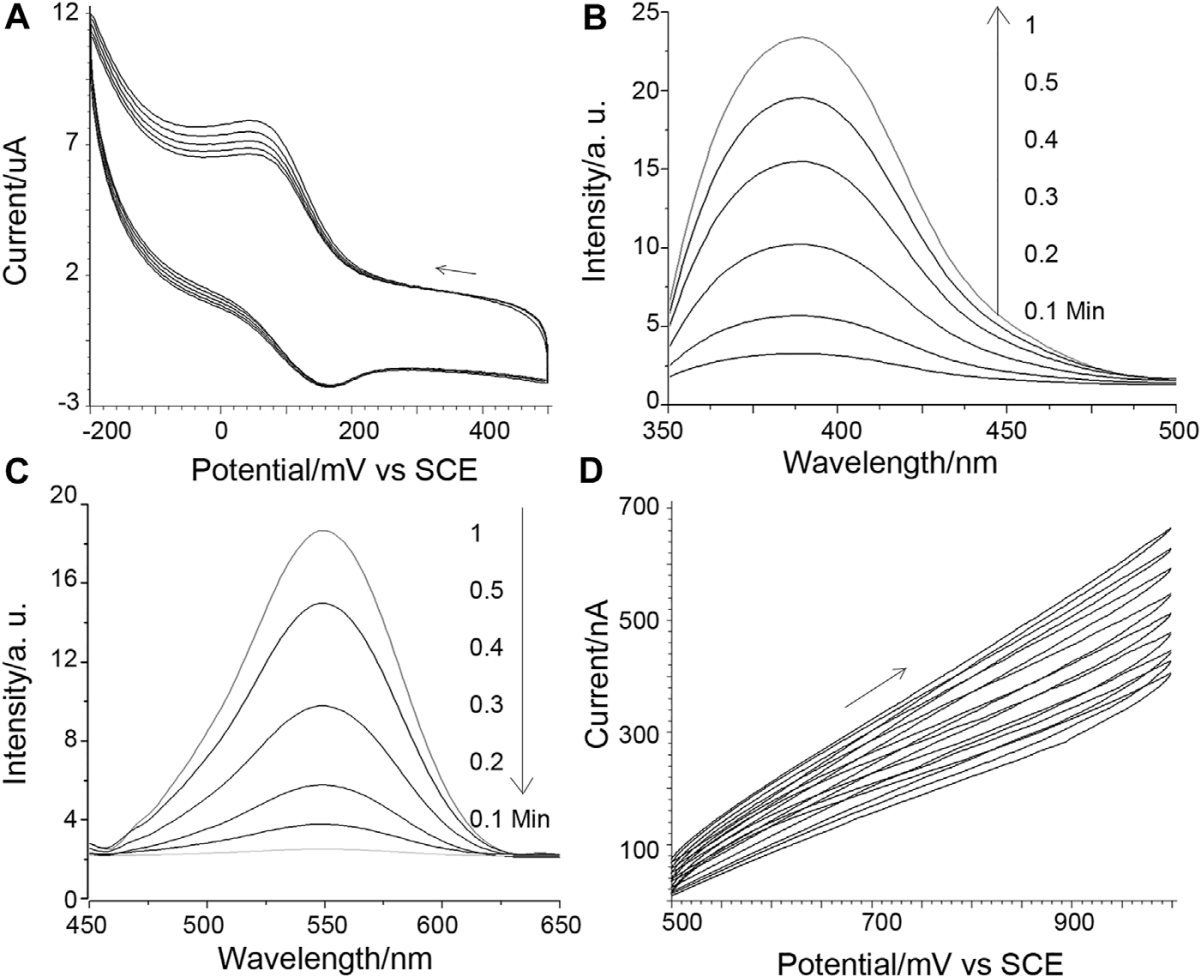
**(A)** Cuion cyclic voltammograms obtained for 10 nM integrin detection. The potential scanning direction is shown by an arrow, and the scan speed is 1 mV/s. **(B)** Di-tyrosine fluorescence measured concurrently with **(A)**: The sensing interface-bearing ITO slide served as the working electrode. A salt bridge setup was employed to connect the reference and counter electrodes to the fluorescence cuvette, which served as the electrolysis cell. **(C)** Tyrosine fluorescence reactions that are parallel. **(D)** Paralleled peroxide evolution, 1 mv/s scan speed.

The designed protein-triggered, conformation-driven, Cu (II) facilitated side-chain di-tyrosine cyclization of the peptide probe, is studied and investigated using various methods. First, possible Cu catalysis is studied using electrochemical methods (Figure 1). Figure 1A shows that when a target protein is detected using the method planned in Scheme 1, a pair of aligned reversible redox oxidation peaks can be seen for the Cu(II) ion complexed by the surface-tethered probe. As the electrochemical scan proceeded, the oxidation peak narrowed and the redox peak correspondingly increased; however, it gradually stopped after mutual reduction of the scan and the original tyrosine (Figures 1B, C). These may suggest that Cu co-promotes electrochemical dityrosine crosslinking: Figure 1A shows how Cu(I) reduced from Cu(II) at the electrode is oxidized back to Cu(II) to catalyze the crosslinking. As a result, there is less and less Cu(I) that can be oxidized by the electrode and more and more Cu(II) waiting to be reduced. Figure 1D shows the development of H_2_O_2_, which is consistent with earlier results for Cu-catalyzed free-radical oxidative bis-tyrosine crosslinking, suggesting that reducing ions may use free radicals to catalyze crosslinking *via* a Fenton-like mechanism (Smith et al., 2007). By measuring and computing the surface density of the tethered probe (Supplementary Figure S3) and substituting a ferrocene tag for the poly-Tyr amplifier, the possibility of inter-probe di-tyrosine cross-linking may be ruled out. Given the short half-life of the radicals produced by the Cu ion and the electrochemical scanning, inter-probe cross-linking can be suppressed if the duration of scanning is controlled.

**FIGURE 2:**
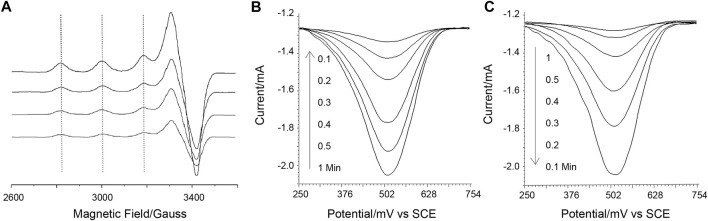
**(A)** X-band electron paramagnetic resonance responses. **(B, C)** is the square wave voltammetric responses of poly-tyrosine detected on the sensing surface and in the solution, sampled at a gradually longer time of electrochemical scanning (as marked on the graphs). A background as studied in Supplementary Figure S5 has been subtracted from the data, and this also applies to the next square wave voltammetric responses of poly-tyrosine.

Then, using X-band electron paramagnetic resonance (X-band EPR), the Cu(II) complex of DAHK, potential Cu catalysis, and DAHK self-cleavage are examined (Figure 2A). Cu(II) may be detected with a typical four-peak separation; g is 2.21; and A has a peak separation of around 184 G (about 570 MHz), showing that the square plane is coordinated with four N-coordinated ligands (Gala et al., 2014). As the electrochemical scan advances, the sampled EPR signal loses significance, while other peaks, such as those identifying Cu(III), grow complicated around 2,000, 3,400, and 4,100 G, are absent (Krebs et al., 1999). These findings could point to self-cleavage of the coordination site, electrochemical reduction of Cu(I) incapable of complexing with the peptide, or both. The electrochemical data mentioned above could point to a decrease of Cu(I) (Figure 1A). The cleavage of polytyrosine may be confirmed by charting the electrochemical response of polytyrosine during the course of the electrochemical scanning process (Figures 2B, C). These two complementary results reveal that the solution response and the surface response vary reciprocally. And this may indicate the oxidative decomposing of the polytyrosine chain, generating the observed signal (Supplementary Figure S4), according to a previous report (Gao and Cranston, 2010).

**FIGURE 3:**
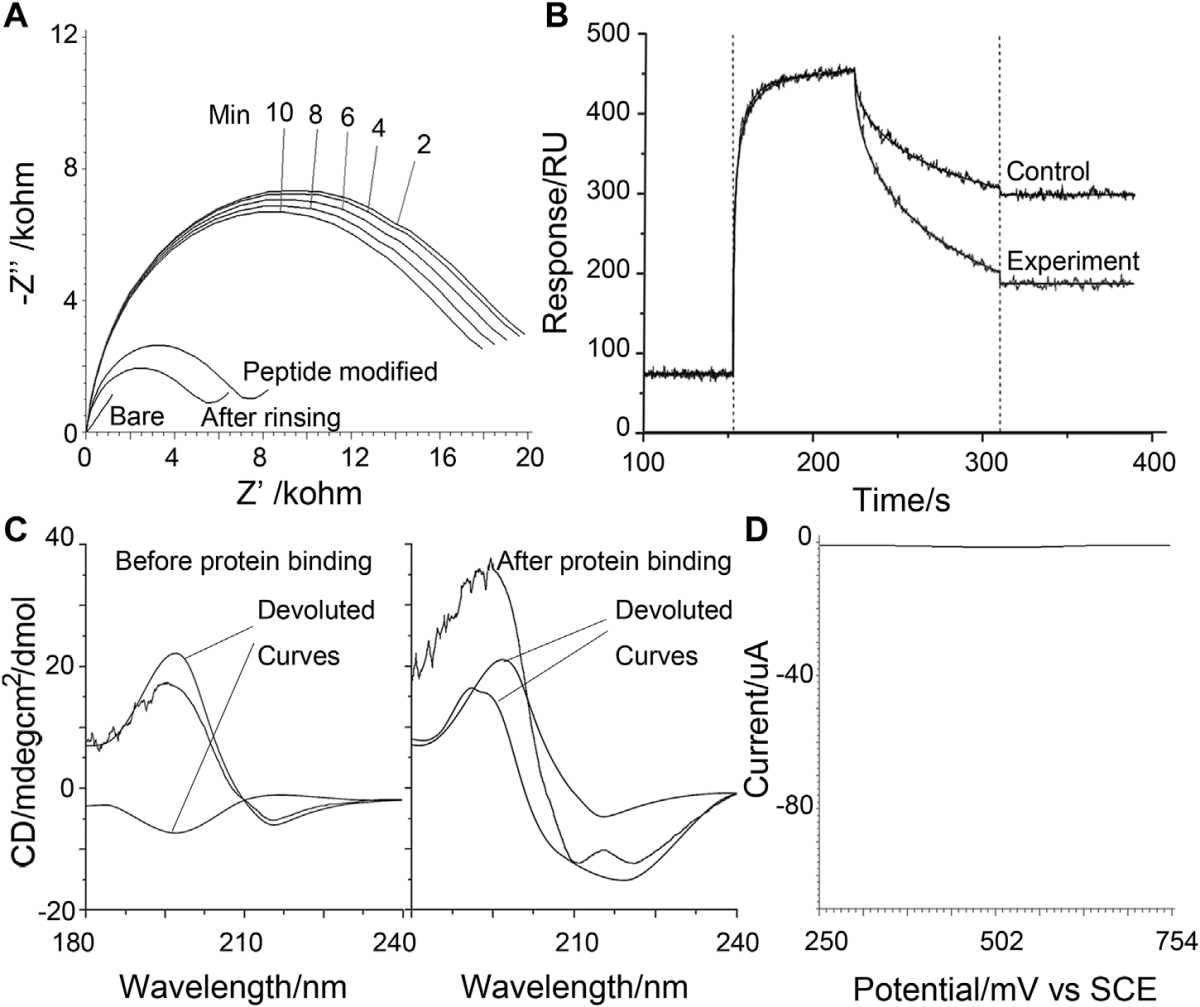
**(A)** Electrochemical impedance spectra recorded under various conditions as labeled on the graph. The time series indicates measurements after a gradually longer duration of electrochemical scanning to agitate the Cu ion facilitated reactions. **(B)** Surface plasma resonance sensorgram contracts the real-time behavior of the self-cleavable probe with a control probe that cannot self-cleave. **(C)** Circular dichroism data was obtained for the control probe before and after protein binding-induced cross-linking. The experimental curve (black line) is de-convoluted to possible components. **(D)** Control square wave voltammograms of poly-tyrosine were obtained in detecting 10 nM MDM2 using a p53-derived alpha-helix forming peptide probe.

Paralleling the above observation, the sensing of the surface is also studied using electrochemical impedance spectra (EIS) and the surface plasma resonance (SPR) method (Figures 3A, B). In the SPR result, a control probe using GHK in the place of DAHK to prevent the self-cleavage (only an N-terminal D can be cleaved shows a shallower slope in the dissociation phase, indicating the existence of self-cleavage for the original probe. Meanwhile, the step-by-step EIS with gradually longer electrochemical scanning time also shows more and more evident losses of impedance, recorded after the violent rinsing; this may also support the above observation of self-cleavage.

Using GHK substituted probes the electrochemical peeled off [by the electrochemical opening of the Au-S bond (Wildt et al., 2010)] from the sensing surface after the violent rinsing step in Scheme 1, and the induced target-induced conformational change can be studied using circular dichroism (CD) (Figure 3C) on the GHK control peptide that cannot self-cleave. In the absence of the target protein, random coil and beta-sheet components are the most evident; with the beta-sheet attributable to the poly-Tyr signal amplifier, the random coil may be assigned to the targeting sequence that represents the most significant length of the rest of the probe. On the contrary, after protein binding and electrochemical scanning, the turn component becomes more evident. Together, these may indicate protein binding induced conformational changes being successfully captured by cross-linking, validating the proposed mechanism. As a control, a peptide derived from p53 to target MDM2, and with a known stiffened *α*-Helix linear form upon binding with MDM2 (Pazgier et al., 2009), can show an almost totally compromised signal response (Figure 3D), confirming the proposed design and its feasibility for the loop and turn conformations that are at least as abundant as the linear helix, as encountered in protein-protein interactions.

In the proposed design, the sample was first incubated with the sensing surface for a time, before the whole sensing system was brought to electrochemical scanning; so both the time for pre-incubation and that for electrochemical scanning have to be optimized. It can be expected that both prolonged incubation and scanning will lead to elevated false positives as the background interference. To suppress this background, prolonged time courses are recorded for both conditions, and contracted with an incorrect positive background time course recorded using BSA as the non-specific control (Supplementary Figure S5). From the resulted time plots, it is clear that the background can rise to evidence after or around the saturation of incubation or scanning is achieved, so a time point slightly before saturation is selected for the two conditions, to succeed as large as possible response with an acceptable background false positive.

**FIGURE 4:**
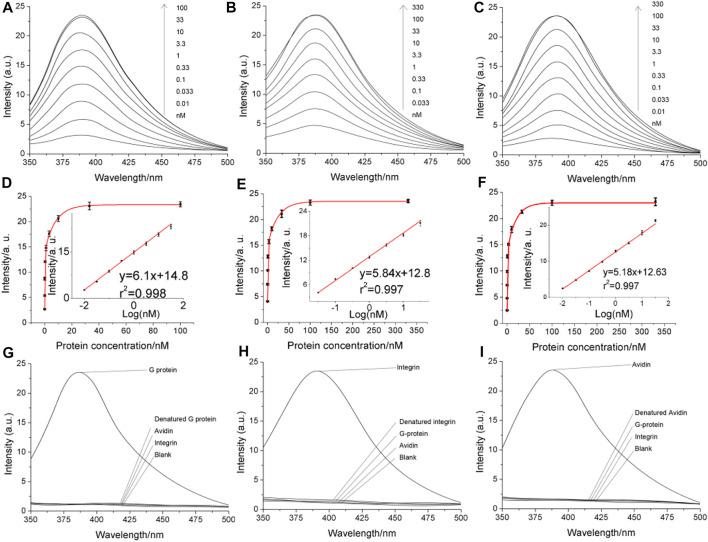
Analytical performance of the proposed method directly using the fluorescent signal of the di-tyrosine product. Panels **(A–C)** are the fluorescent spectra obtained in detecting serially diluted G-protein, integrin and avidin, respectively. The concentrations are marked on the graph. Panels **(D–F)** are quantitative curves obtained by plotting the fluorescent peak signals as a function of the protein concentration. The insets are the linear range. The error bars indicate the standard deviation (*n* = 3). Panels **(G–I)** are the specificity.

**FIGURE 5:**
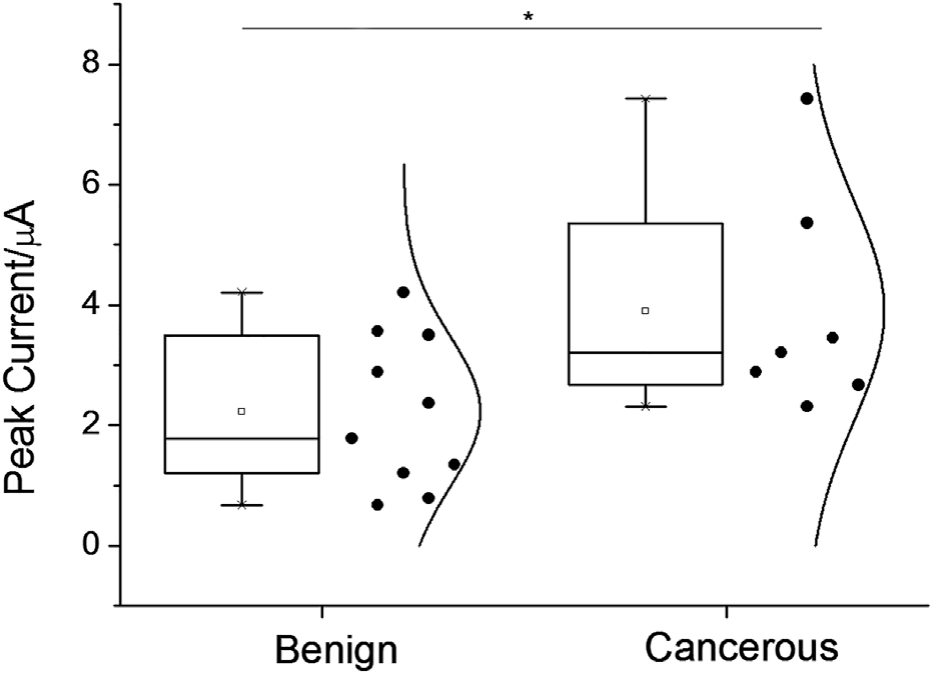
Assay-graded clinical samples’ square-wave voltammetric signal responses were displayed as data distributions for the osteosarcoma condition group and the benign condition group, respectively. Asterisks signify statistical differences of more than 5%.

Quantitative assay performance was assessed in conjunction with the condition optimization discussed above (Figures 4A–F), demonstrating that the fluorescent signal readout grows according to the logarithm of the target concentration, with a limit of detection., also considering the above-selected background level, to be in the range of 1–10 p.m. The specificity is as shown in Figures 4G–I. The electrochemical signal of the polytyroine strand can also be used o quantify the target proteins, as shown in Supplementary Figure S6. A few osteosarcoma cancer tissue samples retrieved during resection and grouped by the TNM staging rule are employed for clinical test. After lysis and fraction, the cellular membrane fraction, containing integrin (α1β1) and used for detection can reveal important group differences between matched paracancerous benign samples and cancer samples (Figure 5). Still, statistically significant differences among stages cannot be observed. The observed elevation of integrin, associated with the development of osteosarcoma cancer, is consistent with previous biomarker report (Boudjadi et al., 2013).

## Discussion

In summary, in light of the complicated nature of protein interactions awaiting biomedical elucidation, we have attempted a highly simplified design using a compact protein-sensing probe that requires only one-step sample incubation before signal collection, and pact the three interconnected biosensing functions of target-binding, signal conversion, and amplification into a small and straightforward peptide molecule. Specifically, a peptide probe containing two tyrosine moieties as two potential covalent bridging sites is designed with the two sites separated by a relatively long protein-targeting sequence; outside of this portion, the linear peptide is extended, at one terminus, by a surface immobilization sequence for tethering onto a sensing surface; and a poly-tyrosine electrochemical oxidative signal amplification sequence, at the other end. The binding of the target protein can lock the peptide in a curved form favoring di-tyrosine cross-linking, At the same time, a DAHK sequence between the two tyrosine bridging sites, and in the vicinity of the protein-targeting sequence, can be electrochemically controlled to cleave itself, resulting in only the protein-bound thus di-tyrosine bridged probe still retaining the amplification sequence. This design may greatly simplify the biosensing scheme and process of biosensor manufacturing, with improved feasibility and robustness towards the field clinical application.

## Data Availability

The original contributions presented in the study are included in the article/Supplementary Material, further inquiries can be directed to the corresponding authors.
