# Supplementary material for: A biocatalytic peptidobiosensing molecular bridge for detecting osteosarcoma marker protein
Source: Front Chem. 2023 Jan 12;10:1112111. doi: 10.3389/fchem.2022.1112111 (PMC9877232; doi:10.3389/fchem.2022.1112111)
Supplement: Supplementary file 1 [file DataSheet1.docx]

**Supporting Information for**

**A Biocatalytic Peptidobiosensing Molecular Bridge for Detecting Osteosarcoma Marker Protein**

Pengwei Jing^a,1^, Ying Wang^b,1^, Weixue Sun^a^, Guishi Li^a^, Zuofu Zhang^a^ ^*^, Qiang Xu^a*^ Hao Li^c*^

*^a^* *Articulation Surgery and Sport Medicine Ward, Yantai Yuhuangding Hospital, 20 East Road of Yu Huangding, Yan Tai,264001, China*

*^b^ Department of Otolaryngology Head and Neck Surgery, Yantai Yuhuangding Hospital, 20 East Road of Yu Huangding，Yan Tai, 264001,China*

*^c^* *School of Biological Science and Technology, University of Jinan, 336 West Road of Nan Xinzhuang,250022, China;*

*Correspondence: zhangzuofu1977@163.com (Zuofu Zhang), xuqiang—830@126.com (Qiang Xu). 2766300864@qq.com (Hao Li)

1. These authors contributed equally to this work


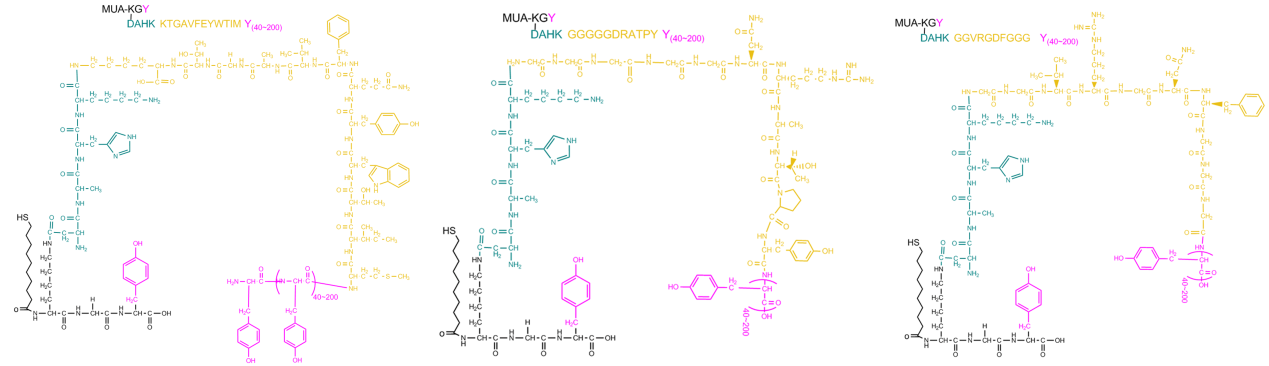


**Figure S1.** Sequences of the three model protein-targeting peptide probes. Left, G-protein; middle, avidin; right, integrin. Different functional motifs are colored corresponding to the color scheme of the probes drawn in Scheme 1.


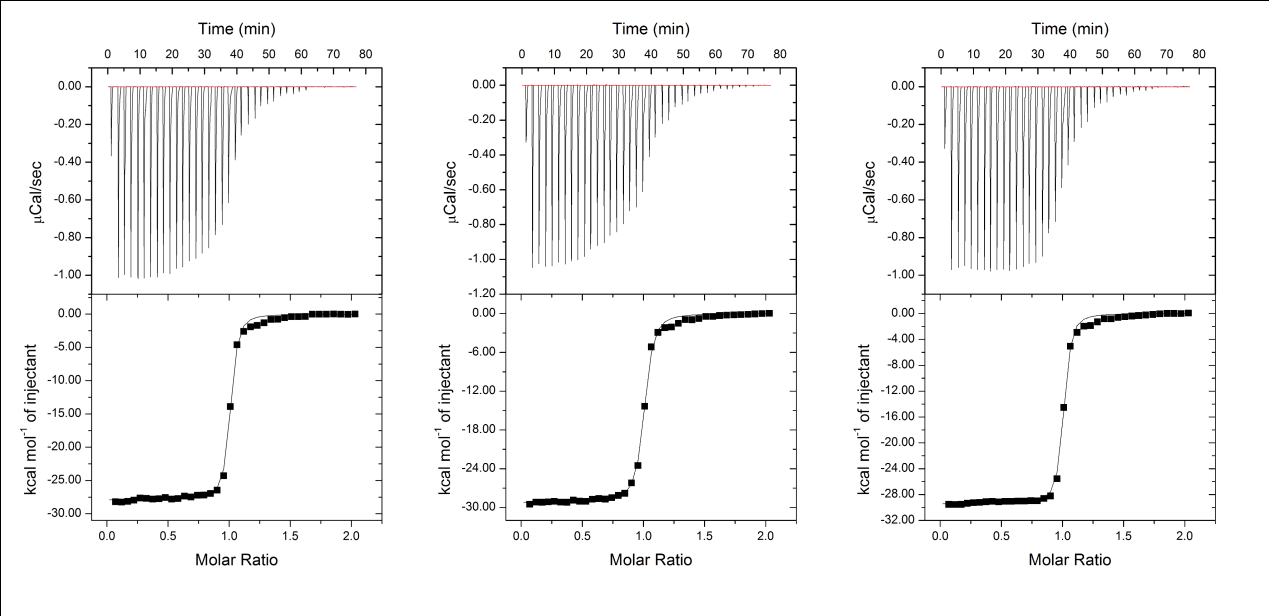


**Figure S2.** Isothermal calorimetric titration of the designed probes into the target protein at 0.5 mM: 0.05 mM concentration ratio, in 10 mM PBS (pH 7.4). From left to right are respectively the result obtained for G-protein, integrin, and avidin. The upper row is the raw data, while the lower shows regression fitting towards a 1 :1 binding model.


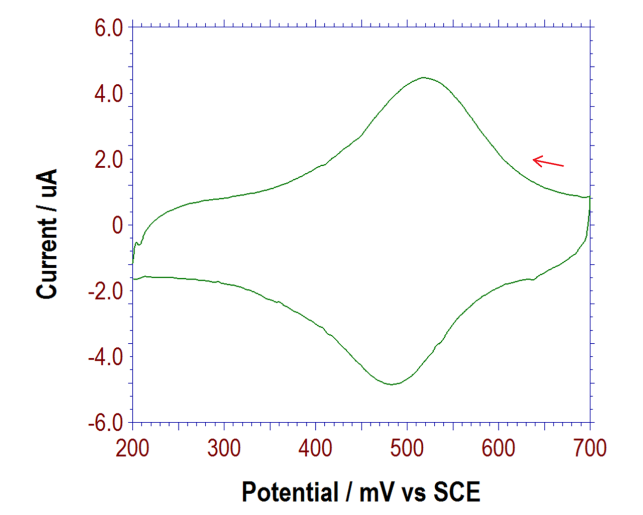
**Figure S3.** Cyclic voltammetric response of a surface tethered ferrocene-tagged probe (for integrin) without the poly-tyrosine motif. The arrow marks the scan direction (scan rate: 1 mV/s). The surface density of the peptide probe immobilized on gold (roughly 10 nM gold membrane deposited on ITO, for this work) could be calculated by Faraday’s laws of electrolysis:

Q=nA_real_ΓF

where n is the number of electrons transferred per ferrocene moiety (n=1); Q is total catholic or anodic charges that flow through the electrode to reduce or oxidize surface ferrocene moiety, which could be derived from dividing either catholic or anodic peak area by scan rate (Above, Q_anodic_=0.00814 ± 0.000839 μC (n=3) ); A_real_ is the real surface area determined from CV catholic peak area of the bare gold surface in H_2_SO_4_ (A_real_= 0.0364 ± 0.0121 cm^2^ (n=3), geometry surface area: 0.03069 cm^2^ ), (however, repeated cyclic voltammetric scanning in H_2_SO_4_ may rapidly remove the gold membrane from the slide).


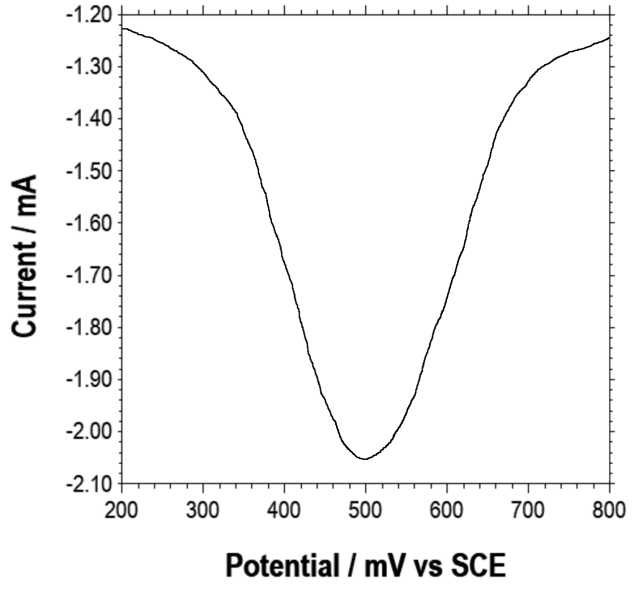


**Figure S4.** Electrochemical signal of poly-tyrosine (of the peptide probe) in the absence of the target protein.

**
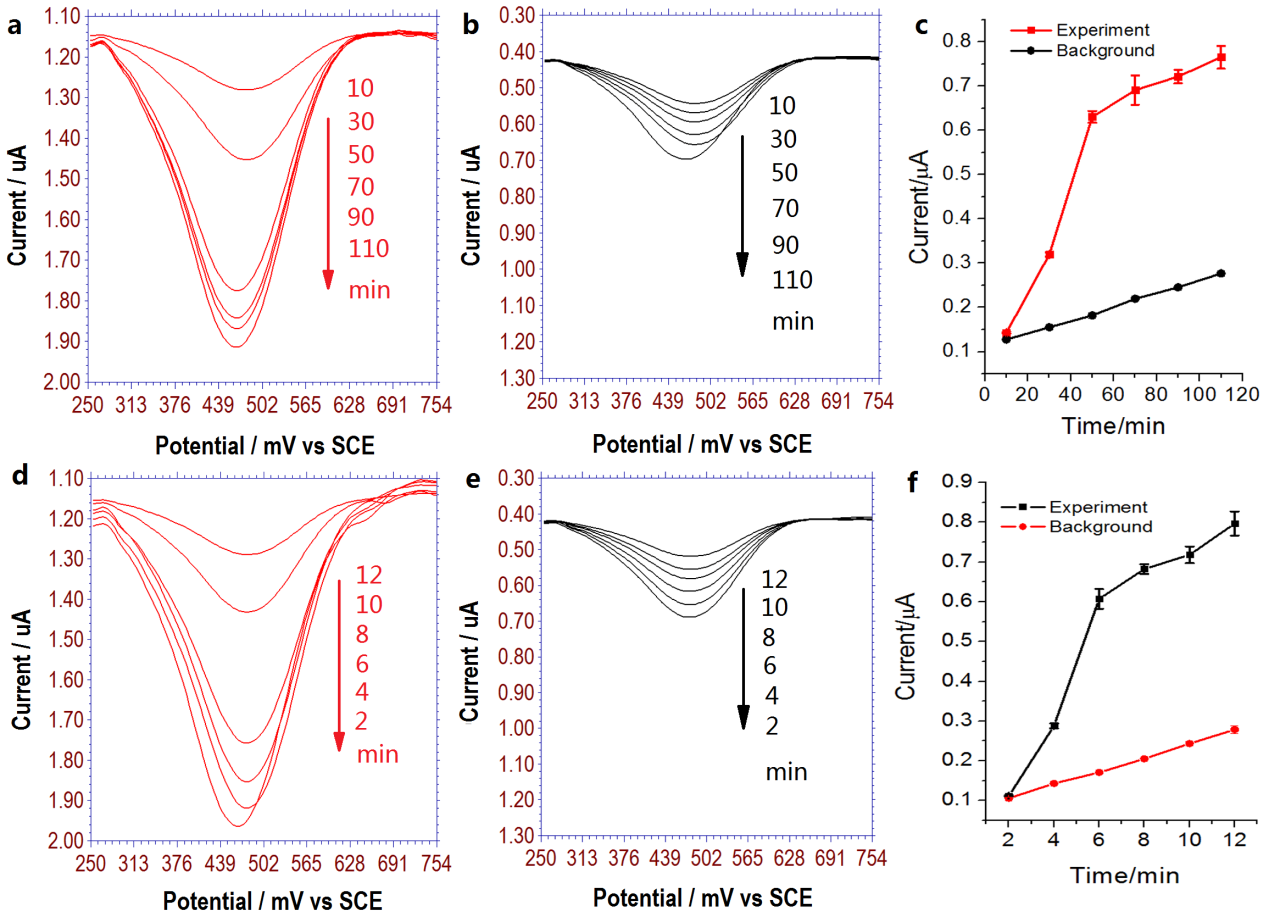
**

**Figure S5.** Optimization of target incubation time and electrochemical cross-linking & cleavage time to minimize the background false positive interference, in detecting 3 nM integrin.

**
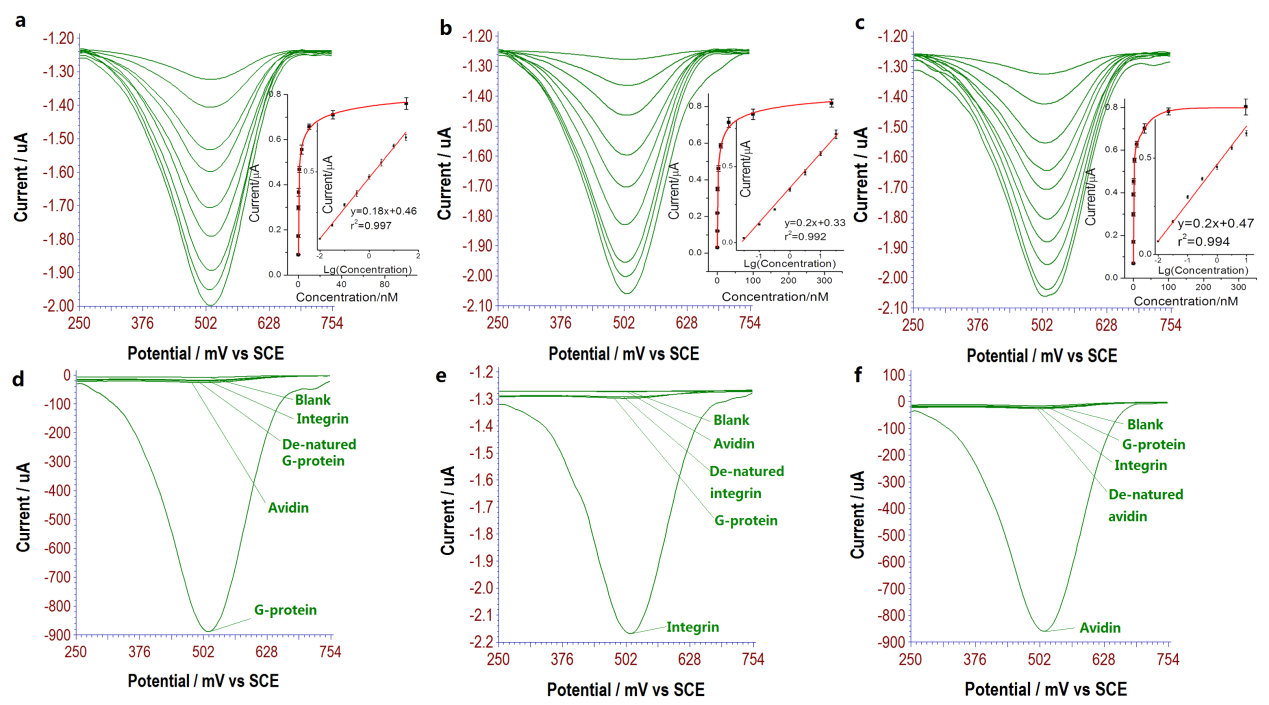
Figure S6.** Performance of the suggested method's analytical tests in identifying G proteins (a, d), integrins (b, e), and avidin (c, f). Square-wave voltammograms of polytyrosine responses at increasing target protein concentrations are shown in the figures insets a through c. Working curves were created by graphing the peak response against target concentration. The error bars (n=3) show the standard deviation. Specificity is indicated by d to f.
